# Therapeutic material basis and underling mechanisms of Shaoyao Decoction-exerted alleviation effects of colitis based on GPX4-regulated ferroptosis in epithelial cells

**DOI:** 10.1186/s13020-022-00652-1

**Published:** 2022-08-16

**Authors:** Juan Li, Xiangge Tian, Jinming Liu, Yuying Mo, Xiaoyi Guo, Yang Qiu, Yuejian Liu, Xiaochi Ma, Yan Wang, Yongjian Xiong

**Affiliations:** 1 Central Laboratory, First Affiliated Hospital of Dalian Medical University, Dalian, 116011 China; 2 Department of Pharmacy, Peking University Shenzhen Hospital, Shenzhen, 518036 China; 3 Pharmaceutical Research Center, Second Affiliated Hospital, Dalian Medical University, Dalian, 116023 China; 4 College of Integrative Medicine, Dalian Medical University, Dalian, 116044 China

**Keywords:** SYD, Ulcerative colitis, Epithelial barrier function, Ferroptosis in epithelial cells, GPX4

## Abstract

**Background:**

Shaoyao Decoction (SYD) is a canonical herbal medicine prescription formulated by Liu Wan-Su in AD 1186. SYD has been widely used to treat inflammatory bowel disease by clearing heat and damp, removing stasis toxin in the intestine; however, the precise mechanisms and therapeutic material basis remain largely unclear. In the present study, we measured the effects of SYD on colitis symptom, epithelial barrier function, epithelial ferroptosis, colonic protein and mRNA expression of glutathione peroxidase 4 (GPX4) in colitis model, and determined whether SYD restored barrier loss in colitis by modulation of GPX4-regulated ferroptosis pathway.

**Methods:**

Colitis was established by infusion with 1 mL 2,4,6-trinitrobenzene sulfonic acid (TNBS) dissolved in ethanol (40% v/v) in rats at a 125 mg/kg dose. Ferroptosis in epithelial cells was determined by flow cytometer. GPX4 promoter-firefly luciferase fusion construct was transfected to Caco-2 cell to determine GPX4 transcription. MS analysis was used to identified ingredients in SYD.

**Results:**

Different doses of SYD significantly alleviated colitis, decreased ferroptosis in epithelial cells, knockout of GPX4 significantly reversed SYD-induced alleviation effects on colitis, restoration of epithelial barrier function, and epithelial ferroptosis. Wogonoside, wogonin, palmatine, paeoniflorin and liquiritin were identified as active ingredients of SYD-exerted alleviation effects of colitis based on GPX4 agonistic transcription.

**Conclusion:**

SYD alleviated chemically induced colitis by activation of GPX4, inhibition of ferroptosis in epithelial cells and further restoration of barrier function. Wogonoside, wogonin, palmatine, paeoniflorin and liquiritin were identified as the key therapeutic material basis of SYD-exerted anti-colitis effects. The findings provide a scientific basis for the therapeutic effect of SYD on colitis.

**Supplementary Information:**

The online version contains supplementary material available at 10.1186/s13020-022-00652-1.

## Introduction

Inflammatory bowel diseases (IBD), composing of ulcerative colitis (UC) and Crohn’s disease (CD), are complex chronic inflammatory conditions with severe diarrhea, abdominal pain, fatigue, and weight loss [1]. In the past decade, inflammatory bowel disease has emerged as a public health challenge worldwide. In North America and Europe, over 1.5 million and 2 million people suffer from the disease, respectively [2]. Thus, it is of great significance to further clarify the pathological mechanism of IBD and explore the novel therapeutic drug.

As previously reported, increased intestinal permeability and disturbed epithelial barrier function represent as important pathological characteristics of IBD [3]. A continuous monolayer of intestinal epithelial cells, which line enteric mucosal surface, represent the first defensive barrier against environmental and microbial attacks by carrying out several critical innate immune functions and maintaining a tight physical barrier. Differentiation of epithelial cells into various cell types ensures the distribution of labour and efficient fulfilment of diverse functions [4]. Dysregulated cell death in epithelium from IBD patients or colitis animals is commonly observed. Genetic and pharmacological perturbation of cell death from experimental IBD models lead to extensive epithelial erosion, leading to a breach of the epithelial barrier, dysbiosis and systemic spread of pathogens [5].

Ferroptosis, a novel non-apoptosis programmed cell death, is caused by the accumulation of oxidized phospholipids, leading to membrane damage and cell lysis [6, 7]. Research showed that ferroptosis participates in pathological condition of cancer, auto-immunology disease, vascular disease and so on [8]. Besides, ferroptosis in colonic epithelial cells from IBD patients was increased than healthy control [9]. Inhibition of lipid peroxidation ameliorates clinical symptoms, improves endoscopic presentations of IBD patients. Ferroptosis in macrophage and intestinal epithelial cells at least partly contributed to increased epithelial cells death and epithelial barrier loss [10].

Glutathione peroxidase 4 (GPX4), a selenoprotein, is the major enzyme catalysing the reduction of phospholipid hydroperoxides in mammalian cells [11]. Lipid hydroperoxides are key intermediates in the lipid peroxidation process, the reduction of lipid hydroperoxides to lipid alcohols requires the catalytic selenocysteine residue of GPX4, and this process prevents iron (Fe^2+^)-dependent formation of toxic lipid reactive oxygen species (ROS) [12]. Therefore, the reduction of GPX4 expression can lead to the accumulation of oxidized phospholipids and lipid peroxidation to promote the occurrence of ferroptosis, which has been demonstrated in animal and clinical experiments [13, 14]. IBD patients’ epithelium exhibit significant reduced GPX4 activity and features of lipid peroxidation, and genetic loss of GPX4 aggravated the clinical outcome of chemically induced colitis [6].

Shaoyao Decoction (SYD) is a canonical herbal medicine prescription formulated by Liu Wan-Su in the Jin-Yuan 92 dynasty (AD 1186) [15], which is made from nine herbs, namely, *Paeonia lactiflora* Pall., *Angelica sinensis* (Oliv.) Diels., *Coptis chinensis* Franch., *Areca catechu* L., *Aucklandia lappa* Decne., *Glycyrrhiza uralensis* Fisch., *Rheum officinale* Baill, *Scutellaria baicalensis* Georgi, and *Cinnamomum tamala* (Buch.-Ham.) T. Nees and Eberm [16]. The plant names have been checked with http://www.theplantlist.org mentioning the data of accessing that website. SYD is commonly used to treat damp-heat dysentery in Chinese traditional medicine, it is mainly used for the prevention and treatment of bacterial dysentery, amebic dysentery, allergic colitis, acute enteritis, and other symptoms associated with the damp-heat syndrome [16]. Recent studies showed that SYD attenuate the DSS-induced inflammatory response in the colon, and LPS-induced RAW264.7 cells at least partly, via the inhibition of both STAT3 and NF-κB signaling pathways [15]. SYD facilitates mucosal repair in colitis model by inhibiting epithelial cell apoptosis, with no effect on proliferation [15, 17, 18]. However, the precise mechanisms and therapeutic material basis of SYD-exerted alleviation of IBD remain largely unclear.

Here, colonic administration of trinitrobenzene sulfonic acid (TNBS) was performed to induced colitis model, effects of SYD on ferroptosis in epithelial cell from colitis model and underlying mechanisms were measured.

## Materials and methods

### Animals

Sprague–Dawley male rats (age 6–8 wk, weighing 200–220 g) were purchased from Liaoning Changsheng Biotechnology Co., Ltd. (Benxi, China). All rats were maintained under specific pathogen-free conditions at Experimental Animal Center, Dalian Medical University (Certificate of Conformity SYXK Liao 2013–0006). The study was conducted following the guidelines of the National Institute of Health Guide for Care and Use of Laboratory Animals (Publication no. 85-23, revised 1985), and was proved by the Dalian Medical University Animal Care and Ethics Committee (No: AEE20047). The animals were acclimatized to laboratory conditions of 23 °C, 12 h/12 h light/dark, and 50% humidity, with ad libitum access to food and water for 2 weeks prior to experimentation. No animals died before the study was started.

### SYD preparation

SYD was made from nine herbs: *Paeonia lactiflora* Pall., *Angelica sinensis* (Oliv.) Diels., *Coptis chinensis* Franch., *Areca catechu* L., *Aucklandia lappa* Decne., *Rheum palmatum* L., *Scutellaria baicalensis* Georgi., *Cinnamomum cassia* Presl., and *Glycyrrhiza uralensis* Fisch., The raw herbs for SYD were purchased from Beijing TongRenTang Co. Ltd. These raw herbs of Shaoyao Decoction were mixed in the ratio of 4:2:2:1:1:1:2:1:1 (dry weight) and then decocted with 10 times volume of distilled water (v/m) for 2 h. The aqueous extracts of SYD were extracted and centrifuged. Supernatant was collected and subjected to condensation under reduced pressure to obtain the semisolid SYD solution [19]. SYD were suspended again in distilled water at a final concentration of 1.2 g/mL [16]. Weight of raw herbs of Shaoyao Decoction (SYD) was 600 g, weight of dry matter of the extracts was 125 g. Thus, extraction yield of SYD was 125 g/600 g × 100% = 20.8%.

### Identification of Shaoyao Decoction using HPLC analysis

The HPLC analysis was performed on a PM1000 series HPLC system (Hitachi, Japan), and the separation was performed on an Innoval ODS-2 column (Agela Technologies, USA, 250 mm × 4.6 mm, 5 μm). Acetonitrile (B)-water (containing 0.03% phosphoric acid Trifluoroacetate) (A) were used as the mobile phase. A gradient elution method was set as follows: 0–50 min, 5–100% B. The volume flow was 0.8 mL/min; the detection wavelength was 230 nm. Before the analysis, the standard mixture of major quality control components including: Ferulic acid, Liquiritin, Berberine hydrochloride, Emodin, Paeoniflorin and Baicalin were prepared as previous reports [20]. Then, both the SYD crude extract and mixed standard were injected for the HPLC analysis. At last, the analysis results were listed in Additional file 1: Fig. S1 which fully confirmed the preparation of Shaoyao Decoction meets the quality requirements.

### Experimental design

Acute colitis was induced according to previously established protocols with slight modifications. Briefly, after a fasting period of 24 h with free access to drinking water, a catheter was inserted through the anus so that its terminus reached approximately to the level of the splenic flexure (8 cm proximal to the anal verge) under urethane anesthesia. Subsequently, the colon was infused with 1 mL TNBS dissolved in ethanol (40% v/v) at a dose of 125 mg/kg [21]. Rats were randomly assigned to five groups of 12 animals each. Control rats in group I were given sterile saline. Group II was a TNBS colitis model control. Groups III, IV, and V were treated by intragastric administration of 150 mg/kg sulfasalazine (SASP), low-dose 4 g/kg SYD, or high-dose 24 g/kg SYD. SASP is an anti-inflammatory drug used to treat IBD [22], and was a positive control for the effects of SYD on colitis. Low dose and high dose of SYD was selected based on its clinical dose for the treatment of IBD and animal equivalent dose [18].

### Shaoyao Decoction medicated serum

The SYD-medicated serum was manufactured according to the previous study [23]. Thirty rats were randomly divided into 3 groups, consisting of control group, low dose SYD (4 g/kg) group and high dose SYD (24 g/kg) group. The rats in the SYD group underwent intragastric administration of SYD two times a day for five consistent days. The rats in the blank serum group received orally administration of physiological saline twice a day for 5 days. At 0.5 h after the final administration, the rats were anesthetized and sacrificed, blood samples were collected from abdominal aorta and centrifuged at 2500 rad/min at 4 °C for 15 min. The serum was isolated and stored for further analysis.

### Monitoring TNBS-induced colitis

Inflammation was evaluated using HE stained colon sections according to previously described morphological criteria. Animal body weights, food intake, rectal bleeding, and diarrhea incidence for each group were recorded daily. The colon was scored for macroscopically visible damage on a 0–10 scale. The disease activity index (DAI) was determined as previously reported [24].

### Measurement of pro-inflammatory cytokines/mediators

In colon, levels of pro-inflammatory cytokines and mediators, including myeloperoxidase (MPO), tumor necrosis factor-alpha (TNF-α), Interleukin-1-beta (IL-1β), Interleukin-6 (IL-6) were determined using double-antibody sandwich ELISAs (R&D Systems, USA), according to the manufacturer’s instructions.

### Measurement of epithelial barrier function

As is previously reported, epithelial barrier function was measured in vivo and in vitro [25]. Rats were denied access to food, but allowed water for 3 h. Then, 22 mL/kg body weight of PBS (pH 7.4) containing 22 mg/mL FITC-dextran were gavaged and serum was harvested 1 h later. Multi-function microplate reader (Thermo Fisher Scientific, Waltham, MA, USA) was used to measure serum recovery of fluorescein isothiocyanate-dextran (FD-4) after SYD administration. An increase in serum recovery of FD-4 indicated the loss of epithelial barrier (see Tables 1, 2).

**Table 1:** Concentrations of five active ingredients in plasma and colon samples post SYD administration (ng/mL)

	Plasma sample (0.5 h)	Colon sample (0.5 h)	Plasma sample (2 h)	Colon sample (2 h)
Liquiritin	1.39	1.45	1.29	1.34
Paeoniflorin	102	79.6	42.5	113
Palmatine	10	2.94		14.8
Wogonoside	79.5	5	37.2	1.97
Wogonin	0.947	40.8	2.99	51.6

**Table 2:** Ion-pair selection method used in MS

	ESI Model	Q1	Q3
Palmatine	Positive	352.1	336.1
Wogonin	Positive	285.3	270.1
Liquiritin	Negative	417.0	255.0
Wogonoside	Negative	459.0	283.0
Paeoniflorin	Negative	525.0	525.0

Epithelial barrier loss dysfunction model in vitro was established using Caco-2 cells (1 × 106) monolayer incubated LPS (0.5 ng/mL) for 3 days [26]. Caco-2 monolayers were treated with LPS or Shaoyao Decoction medicated serum. Caco-2 monolayers without drug treatment served as normal control. Epithelial voltohmmeter was used to determine transepithelial electrical resistance (TEER) of Caco-2-plated filters. Decreased level of TEER implicated an increase in monolayer permeability and epithelial barrier loss in vitro.

### Transmission electron microscopy

Rats were harvested and colonic tissue was fixed with 2.5% glutaraldehyde, followed by post-fixation in 2% osmium tetroxide with 1.6% potassium ferrocyanide in 0.1 mol/L sodium cacodylate. Colonic tissues were cut into 5-mm^3^ samples, stained with 2% uranyl acetate, dehydrated in ethanol, and embedded in eponate. Semi-thin Sects. (80 nm) were stained with hematoxylin and eosin, 2% uranyl acetate and lead citrate. Images were captured with a Hitachi H7600 TEM in the microscope core [23].

### Flow cytometry analysis

Caco-2 cells were incubated with BODIPY 581/591 C11 (5 μM) at 37 °C in cell incubator for 30 min. Cells were subsequently washed, resuspended in PBS and transferred through a cell strainer for flow cytometry (The BD Accuri™ C6 Plus) [9].

### Small interference RNA-target silencing of GPX4

GPX4 siRNAs were introduced into Caco-2 cell line at a concentration of 30 nmol/L by transient transfection with Lipo2000 Transfection Reagent, following guidelines provided by manufacturer. At 48-h post-transfection, the cells were collected for further studies. The effect of siRNA was evaluated by measuring the expression of GPX4 [27].

### Luciferase assay

Caco-2 cells were co-transfected at day 2 with GPx4 promoter-driven luciferase fusion construct (pGL3-GPX4) and Renilla luciferase expression plasmid pRL-TK together with Lipo 3000 (Invitrogen) transfection reagent in serum-free medium. The promoter luciferase vector pGL3 basic was co-transfected with pRL-TK in control experiments for the determination of background [28].

### Identification of ingredients of Shaoyao Decoction using MS analysis

Wogonoside, Paeoniflorin, Baicalin, Emodin, Liquiritin, Baicalein, Ferulic acid, Cinnamic acid, Gallic acid, Palmatine, Coptisine, Berberine, Dehydrocostus Lactone, Wogonin, Imperatorin, Arecoline, Coumarin were mixed at a concentration of 50 ng/mL. Chromatographic separation with MS was achieved using a programmed gradient mobile phase consisting of (A) 0.1% formic acid in water and (B) acetonitrile. The flow rate used was 0.3 mL/min and the injection volume was 4 μL for all the analyses [10].

### Macromolecular docking

We molecularly docked five monomers (wogonoside, wogonin, palmatine, paeoniflorin and liquiritin) with GPX4. The small molecule structures were retrieved from the NCBI PubChem database (https://pubchem.ncbi.nlm.nih.gov) [29], and the Compound CIDs were 3,084,961, 5,281,703, 19,009, 442,534, 503,737. The 3D structure of GPX4 (PDB code: 5l71) was obtained from The Protein Data Bank (PDB; http://www.rcsb.org/pdb/) [30]. Ligand tools in Autodock4 are used to prepare ligands and receptors for AutoGrid, including removing existing ligands and crystal water from the target protein, adding hydrogen bonds and partial charges to small molecules, and identifying rotatable bonds that will be explored during docking. Then run autodock for automated molecular docking. The binding energy between the ligand and receptor is then obtained. The lower the value, the more stable the docking result. Finally, use PyMOL for graphing.

### Statistical analysis

The animal experiments, in vitro experiments, and data analysis were conducted according to a single-blind study design. One-way analysis of variance was used for multiple group comparisons; multiple comparison between the groups was performed using S–N–K method. Two-tailed unpaired Student’s *t*-test was used to evaluate between two group comparisons. Data were expressed as means ± SD. The data were a normally distributed and the groups had equal variances. All experiments were repeated for at least three times. P-values less than 0.05 were considered statistically significant.

## Results

### Alleviation effects of SYD on colitis symptom

**Fig. 1:**
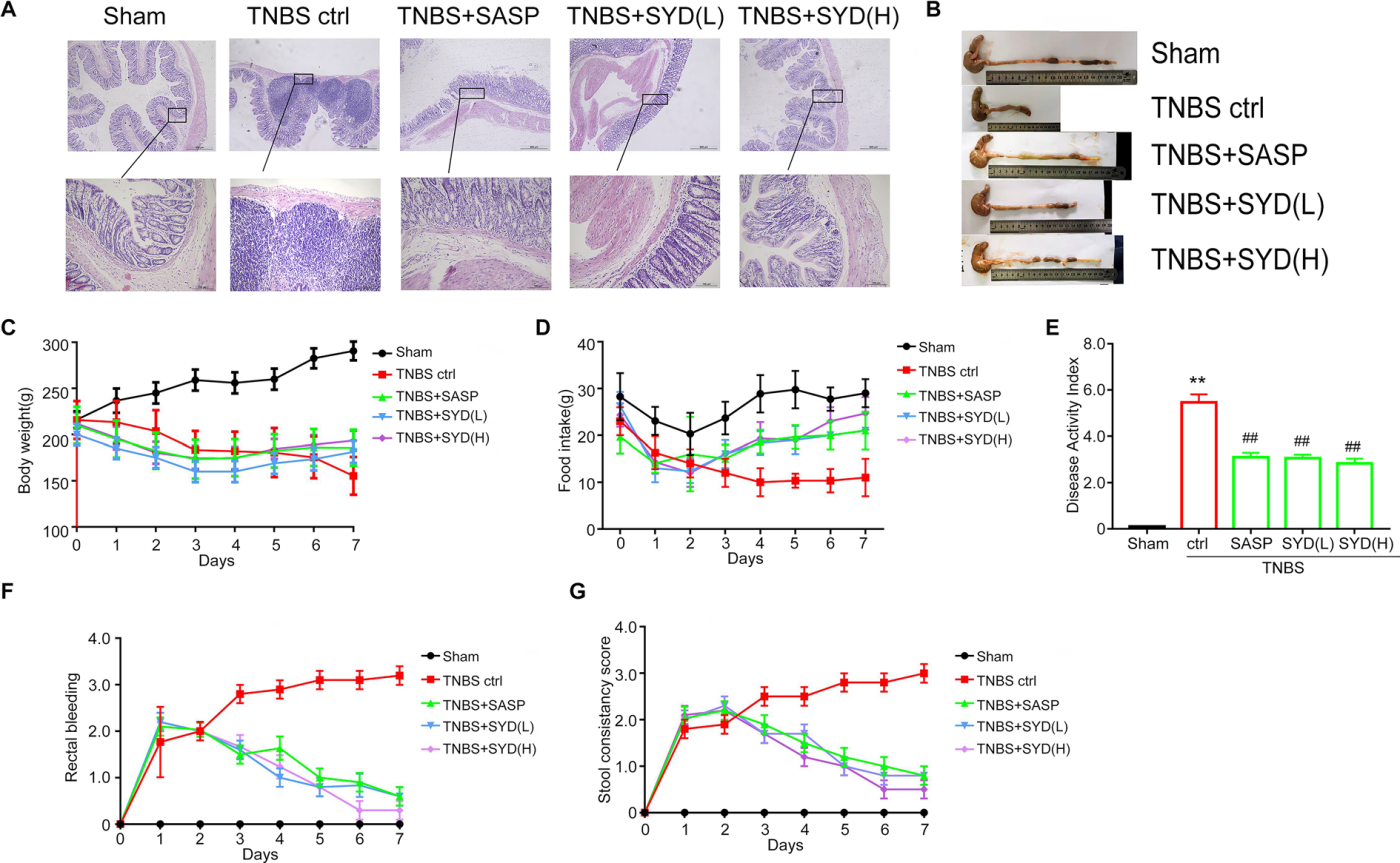
Alleviation effects of Shaoyao Decoction (SYD) on 2,4,6-trinitrobenzene sulfonic acid (TNBS)-induced colitis in rats. Rats was colonic administrated with TNBS to induce colitis model. **A** hematoxylin and eosin (HE)-staining analysis of rats colonic tissue was performed. Effects of SYD on **B** colon length, **C** body weight, **D** food intake, **E** disease activity index, **F** rectal bleeding score, and **G** stool consistency score in rats with colitis were measured. Data are expressed as the mean ± SD. ^**^P < 0.01 compared with the sham group (n = 6); ^##^P < 0.01 compared with the TNBS control group (n = 7). SASP, sulfasalazine; SYD (L), 4 g/kg SYD; SYD (H), 24 g/kg SYD

As shown in Fig. 1, TNBS-administrated rats showed excessive intestinal inflammation, which was characterized by developed body weight loss (Fig. 1C), food intake loss (Fig. 1D), rectal bleeding (Fig. 1F), diarrhea (Fig. 1G) and high disease activity index (DAI) score (Fig. 1E). Colon length and histology study showed that TNBS-treated rats exhibited atrophy of intestinal villi, infiltration of inflammatory cells (Fig. 1A), decrease in colon length (Fig. 1B). All these data confirmed the successful establishment of colitis model. Different dose of SYD and SASP treatment alleviated rats colitis symptom, including increase in body weight/food intake, alleviation of diarrhea and rectal bleeding, decrease in DAI score, restoration of intestinal villi, reduce of inflammatory cells infiltration, increase in colon length. Together, these data showed that SYD significantly alleviated colitis symptom induced by TNBS.

### SYD-exerted inhibition of colonic inflammation and restoration of epithelial barrier function

**Fig. 2:**
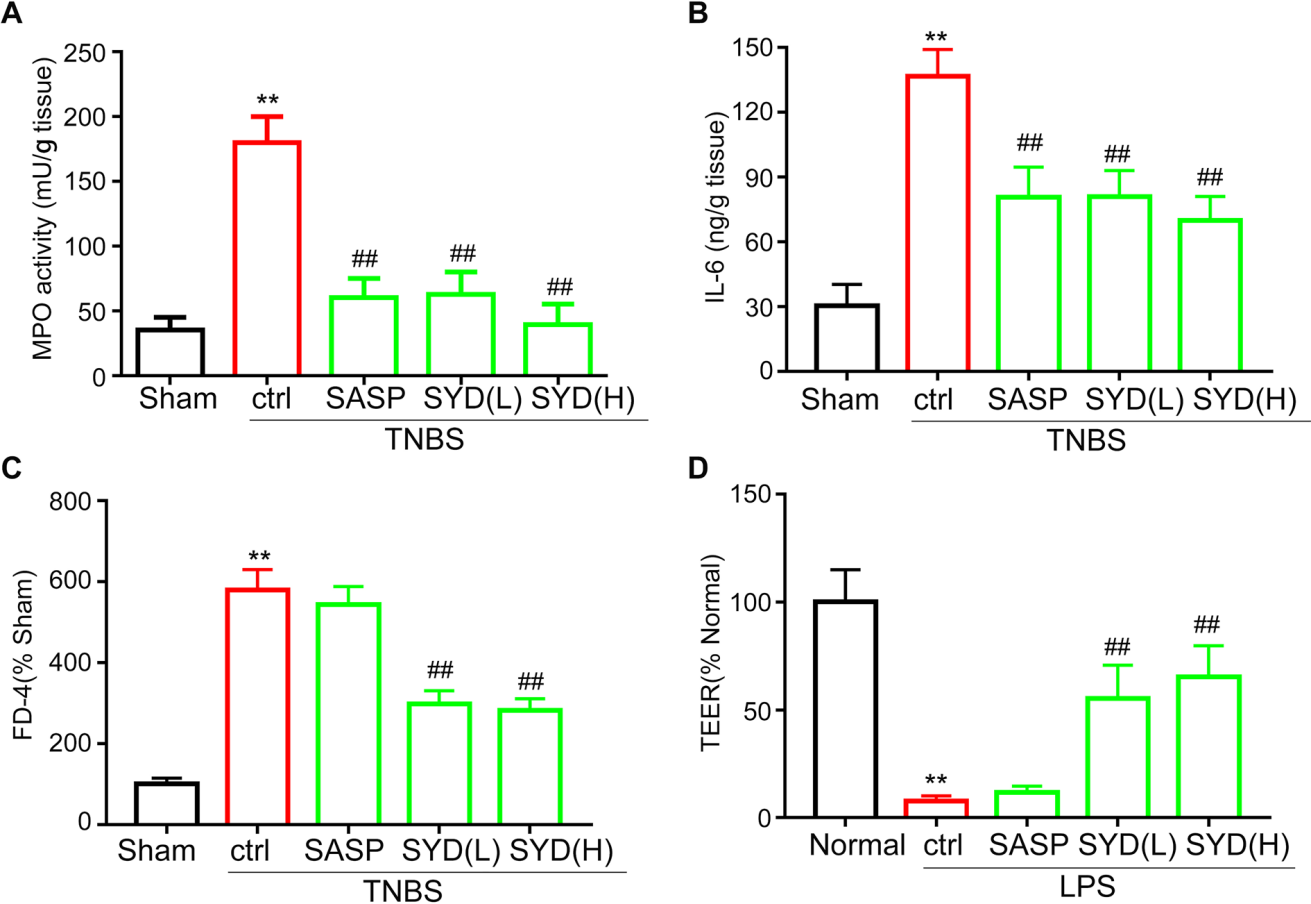
Effects of Shaoyao Decoction (SYD) on colonic inflammation and epithelial barrier function in colitis rats. Rats with colitis were gavaged with SYD (4 g/kg/day; 24 g/kg/day) for 7 days, then **A** Myeloperoxidase (MPO) activity, **B** colonic pro-inflammatory cytokine IL-6 and **C** serum fluorescein isothiocyanate-dextran (FD-4) level were measured. Transepithelial electric resistance (TEER) was measured to demonstrate the effects of SYD on barrier function in lipopolysaccharide (LPS)-induced barrier loss model in vitro (**D**). ^**^P < 0.01 compared with the sham group (n = 7); ^##^P < 0.01 compared with 2,4,6-trinitrobenzene sulfonic acid (TNBS)/LPS control group, respectively (n = 7)

The enhancement of neutrophil infiltration and levels of proinflammatory cytokines and mediators are associated with the initiation of intestinal inflammation [31]. MPO activity and levels of proinflammatory cytokines (IL-6, IL-1β, TNF-α) in colonic tissues from colitic rats were significantly increased compared to those in the control rats. SYD administration reversed the increased MPO activity and changes in proinflammatory cytokines levels (Fig. 2A, B). These data showed that SYD exerted significant anti-inflammation effects in colitis rats.

Caco-2 monolayer incubated with LPS (0.5 ng/mL) for 3 days was used to mimic epithelial barrier loss in vitro. Serum recovery of FD-4 and TEER level of Caco-2 monolayer were measured in vivo and in vitro, respectively. And serum FD-4 level (Fig. 2C) were significant higher in colitis rats than that in normal ones, TEER level (Fig. 2D) was lower in LPS treatment group (TEER value: 115 ± 10) than in control group (TEER value: 520 ± 15); these data implicating epithelial barrier loss in colitis model group. SYD (TEER value: 236 ± 11 for low dose, 287 ± 16 for high dose), but not SASP (TEER value:126 ± 13), administration increased TEER level in LPS-treated group; serum FD-4 level were significantly decreased after SYD treatment in vivo. All these showed that SYD supplementation restored epithelial barrier dysfunction in colitis rats.

### SYD-exerted inhibition of ferroptosis in epithelial cells

Literatures showed that increased ferroptosis in epithelial cells from IBD patients was observed as compared with healthy control [9], and stimulation of ferroptosis led to increased intestinal inflammation and epithelial barrier loss in colitis model [22]. Here we measure whether inhibition of ferroptosis contributes to SYD-exerted alleviation effects of colitis.

**Fig. 3:**
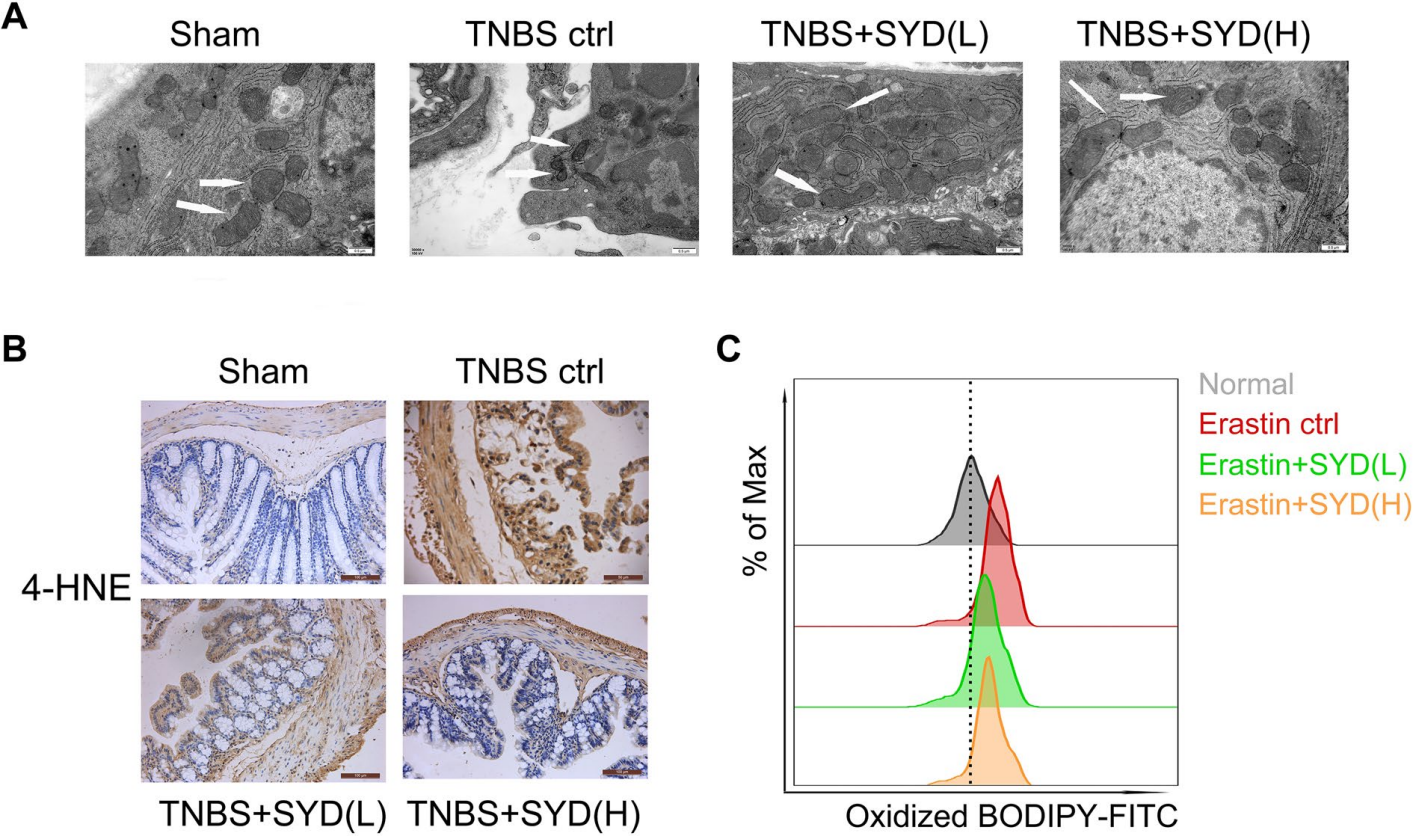
Shaoyao Decoction (SYD) inhibited ferroptosis in colonic epithelial cells from colitis rats. **A** Mitochondrial morphology in colonic epithelial cells from colitis rats was determined using transmission electron microscopy (TEM); **B** lipid peroxidation product 4-HNE expression was measured to reflect lipid peroxidation rate. Besides, **C** effects of SYD-medicated serum on lipid peroxidation were also measured in Caco-2 cells pretreated with ferroptosis inducer erastin. ^**^P < 0.01 compared with the sham group (n = 7); ^##^P < 0.01 compared with 2,4,6-trinitrobenzene sulfonic acid (TNBS) control group, respectively (n = 7)

Transmission electron microscopy (TEM) results (Fig. 3A) suggested pyknosis of mitochondrial in epithelial cells in colonic tissues from colitis rats. Elevated iron level and increased expression of 4-HNE were also confirmed in colon tissues from colitis rats (Fig. 3A), implicating increased ferroptosis in epithelial cells of colitis rats. Caco-2 cells incubated with ferroptosis inducer erastin were selected to measure the effects of SYD on ferroptosis in epithelial cells. Shaoyao Decoction medicated serum administration significantly suppressed ferroptosis in colonic epithelial cell in colitis rats, including restoration of mitochondrial morphology (Fig. 3A), decreased expression of 4-HNE (Fig. 3B) and reducing lipid peroxidation (Fig. 3C).

### SYD induced activation of GPX4 transcription and increase of protein expression

**Fig. 4:**
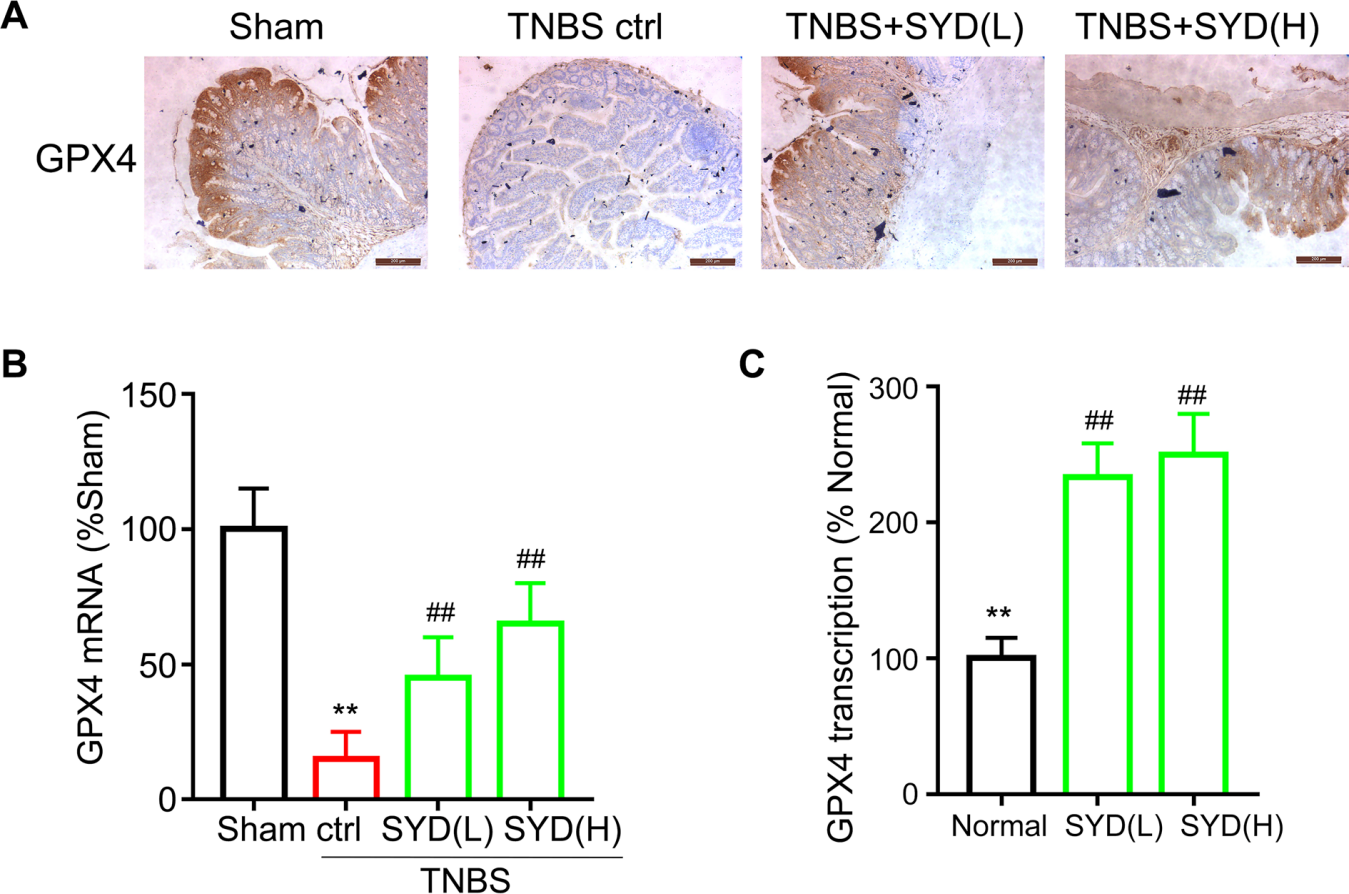
Shaoyao Decoction (SYD) increased glutathione peroxidase 4 (GPX4) expression in colonic tissues from colitis rats and proved GPX4 transcription. Rats with colitis were gavaged with SYD for 9 days, then colonic protein and mRNA expressions of GPX4 were measured in different groups using immunology blot (**A**) and qPCR (**B**). Luciferase assay (**C**) was performed to measure the transcription of GPX4 gene in different groups

Ferroptosis is a newly described form of regulated cell death, defined as the result of missing or insufficient activity of the selenoperoxidase Glutathione Peroxidase 4 (GPX4), which causes a specific form of cell death operated by membrane lipid peroxidation. Here we measured the effects of SYD on mRNA and protein expressions of colonic GPX4 from TNBS treated rats. Immunohistochemistry results (Fig. 4A) showed that colonic GPX4 expressions was significantly decreased after TNBS treatment, which could be reversed by SYD. Besides, SYD also increased GPX4 mRNA level in colitis rats (Fig. 4B), indicating that SYD may increase GPX4 expression by modulating transcription of GPX4 gene. Thus, GPX4 promotor luciferase assay was performed, results (Fig. 4C) proved that different doses of Shaoyao Decoction medicated serum both increased transcriptional activity of GPX4.

### Knockdown of GPX4 significantly abolished SYD-exerted alleviation of colitis

**Fig. 5:**
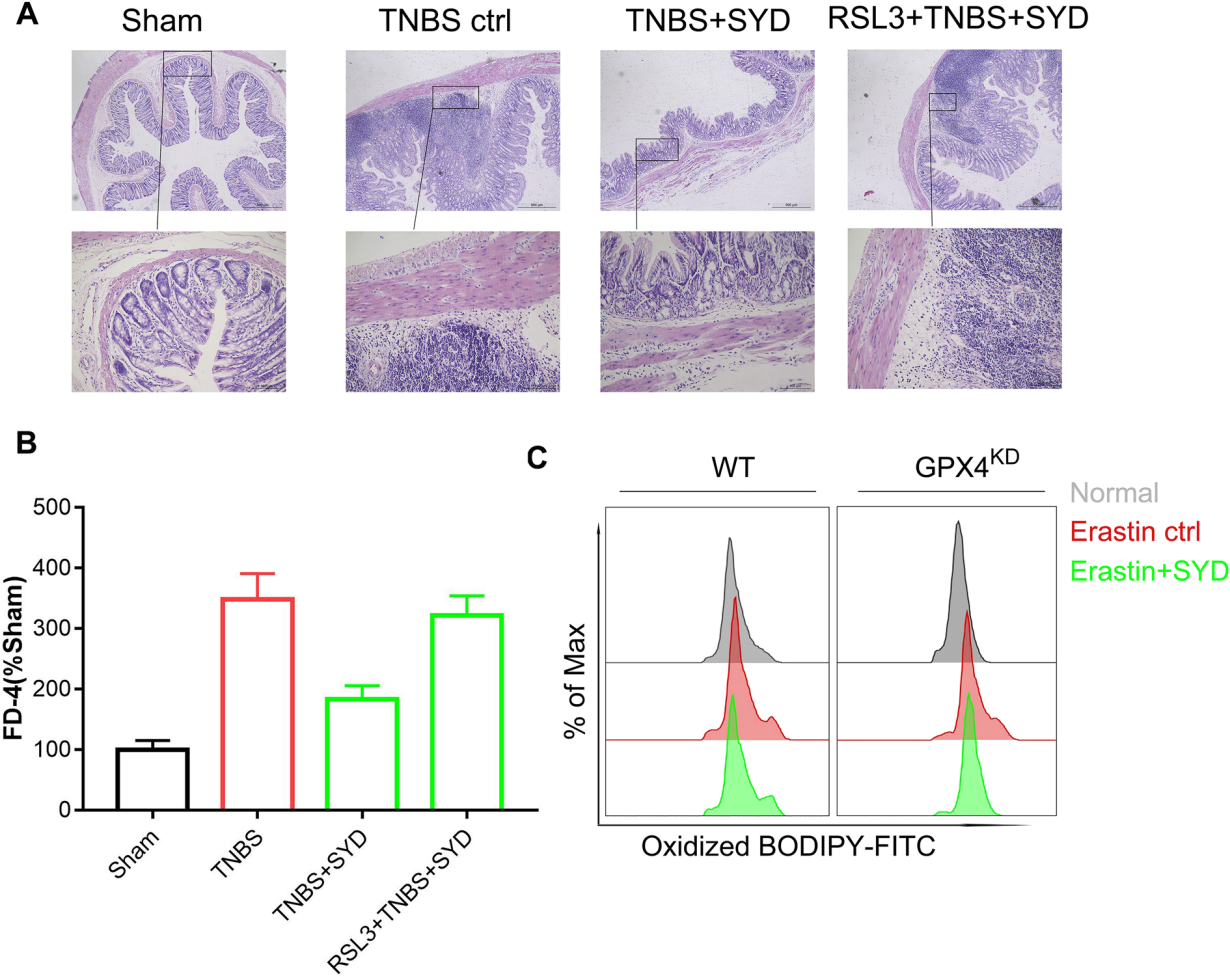
Pharmacological inhibition or genetic loss of glutathione peroxidase 4 (GPX4) significantly abolished Shaoyao Decoction (SYD)-induced alleviation of colitis and inhibition of ferroptosis. Rats were gavaged with GPX4 inhibitor RSL3 for 3 days before TNBS administration. HE-staining analysis of inflammation (**A**) and serum fluorescein isothiocyanate-dextran (FD-4) content (**B**) were determined. Si-RNA-mediated gene silencing was performed to mimic knockdown of GPX4 in vitro, lipid peroxidation (**C**) in erastin-treated colonic epithelial cells were determined using flow cytometer. ^**^P < 0.01 compared with the normal group (n = 7); ^##^P < 0.01 compared with 2,4,6-trinitrobenzene sulfonic acid (TNBS) treated control group, respectively (n = 7)

To verify whether GPX4 gene is the target for SYD-induced alleviation of colitis. GPX4 specific inhibitor RSL3 were purchased and knockdown of GPX4 using siRNA gene silencing technology in Caco-2 cells were established. Results (Fig. 5) showed that compared with sham group, pharmacological inhibition of GPX4 significantly reversed SYD-induced alleviation of colitis system and restoration of barrier dysfunction in colitis rats. Further, knockdown of GPX4 in vitro largely abolished SYD-induced inhibition of ferroptosis in epithelial cells. These data indicated that SYD exerted alleviation of colitis in a GPX4-dependent manner.

### Identified of ingredients in SYD based on GPX4 agonistic activity

**Fig. 6:**
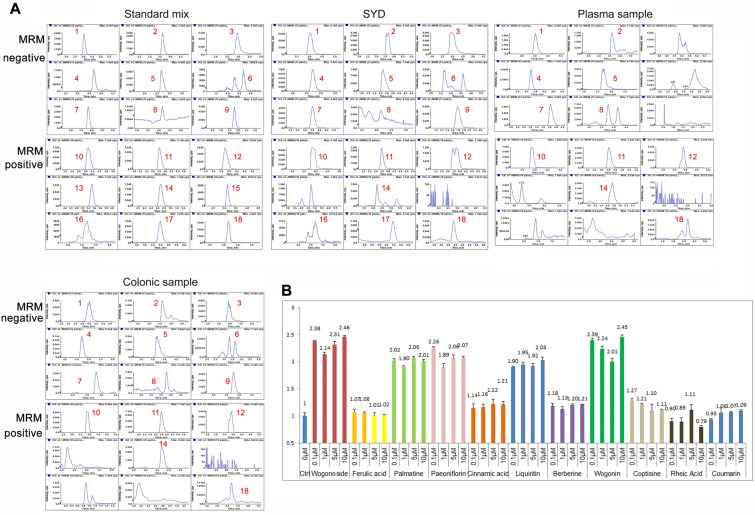
Material basis of Shaoyao Decoction (SYD)-exerted anti-colitis effects. **A** Identification of ingredients in SYD that exerted alleviation effects using MS. **B** Modulation of ingredients in SYD on glutathione peroxidase 4 (GPX4) transcription using Luciferase assay. 1. Wogonoside; 2. Paeoniflorin; 3. Baicalin; 4. Emodin; 5. Liquiritin; 6. Baicalein; 7. Ferulic acid; 8. Cinnamic acid; 9. Gallic acid; 10. Palmatine; 11. Coptisine; 12. Berberine; 13. Costundide; 14. Wogonin; 15. Imperatorin; 16. Arecoline; 17. Dehydrocostus Lactone; 18. Coumarin

0.5 h after SYD orally administration, MS assay was performed to measure the ingredients in plasma and colon samples from colitis rat. Eighteen standard substances were mixed at the concentration of 50 ng/mL. MS analysis results (Fig. 6A) showed that 16 ingredients were confirmed in SYD; 11 ingredients and 14 ingredients were identified in rat plasma and colon samples, respectively. Therefore, 11 ingredients were identified as the active ingredients in SYD.

Modulation of ingredients in SYD on GPX4 transcription was then measured. Luciferase results (Fig. 6B) showed that wogonoside, wogonin, palmatine, paeoniflorin and liquiritin showed more significant activation effects of GPX4 transcriptional activity, compared with other ingredients.

### Modulation of SYD ingredients on epithelial barrier function and epithelial ferroptosis

**Fig. 7:**
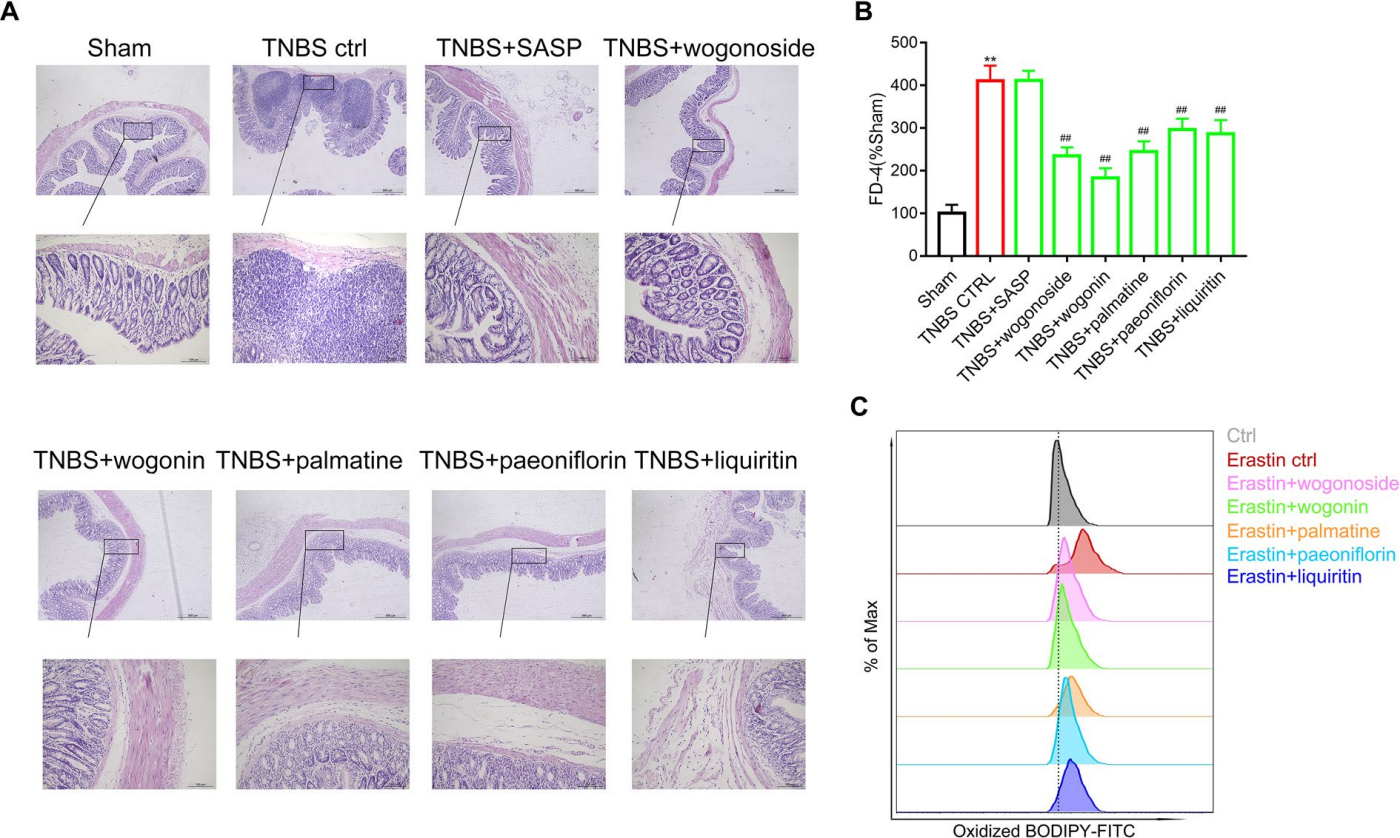
Effects of ingredients in Shaoyao Decoction (SYD) on colitis and epithelial ferroptosis. Rats was colonic administrated with 2,4,6-trinitrobenzene sulfonic acid (TNBS) to induce colitis model, **A** hematoxylin and eosin (HE)-staining analysis of rats’ colonic tissue was performed. Effects of wogonoside, wogonin, palmatine, paeoniflorin and liquiritin on serum fluorescein isothiocyanate-dextran (FD-4) contents (**B**) were measured. Besides, effects of ingredients in SYD on epithelial ferroptosis (**C**) were measured using flow cytometer

Effects of SYD ingredients on colitis and epithelial ferroptosis were then verified. HE staining results (Fig. 7A) showed that all the five ingredients alleviated colitis symptoms, including restoration of intestinal villi, and reduce of inflammatory cells infiltration. Multifunctional enzyme marker results (Fig. 7B) showed that all the five ingredients decreased serum FD-4 level in TNBS-treated rats, implicating restoration of epithelial barrier function. Flow cytometer results (Fig. 7C) showed that all the five ingredients in SYD decreased the lipid peroxidation level induced by erastin.

Taken together, wogonoside, wogonin palmatine, paeoniflorin and liquiritin was identified as the key material basis of SYD-induced alleviation on colitis based on GPX4 agonistic activity.

### Molecular interaction between the key active ingredients of SYD and GPX4

**Fig. 8:**
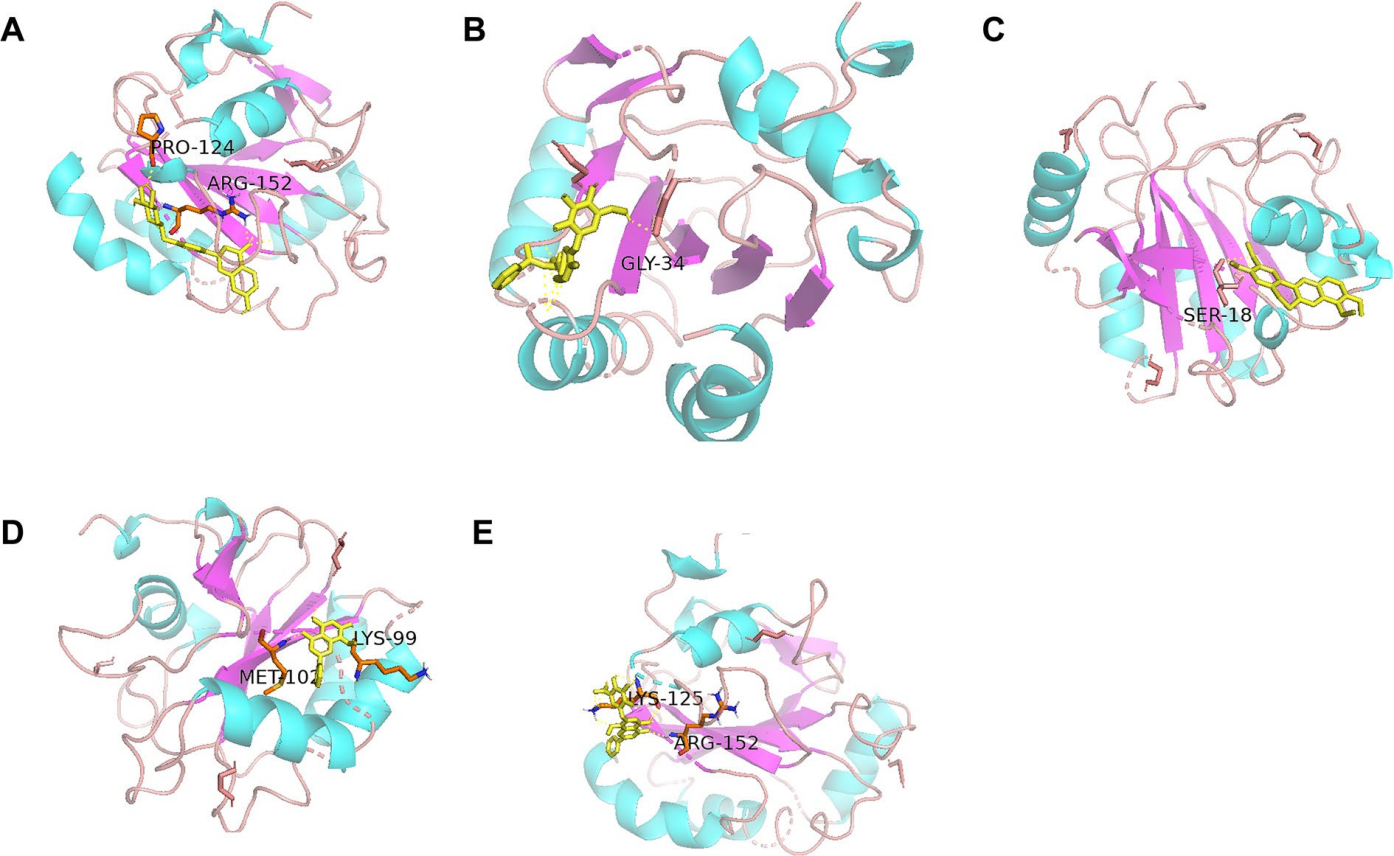
Molecular interaction between the key active ingredients of SYD and GPX4. Interaction between **A** wogonoside, **B** wogonin, **C** palmatine, **D** paeoniflorin, **E** liquiritin and GPX4 by macromolecular docking methods

Molecular docking experiment was performed to investigate the activation mechanism between key active ingredients of SYD and GPX4. Results showed that key active ingredients of SYD could bind to GPX4. As shown in Fig. 8, liquiritin, paeoniflorin, palmatine, wogonoside, wogonin formed hydrogen bonds with amino acid residues Pro124 and Arg152; Gly34; Ser18; Lys125 and Arg152; Met102 and Lys99 respectively in GPX4.

## Discussion

In the present study, we measured the effects of SYD on TNBS-induced colitis model in rats. SYD exerted significant alleviation effects of colitis, repression of inflammation and restoration of epithelial barrier function based on GPX4-regulated ferroptosis.

SYD has been widely used to treat IBD by clearing heat and damp, removing stasis toxin in the intestine [18], but the precise mechanisms and therapeutic material basis remain largely unclear. Increased epithelial barrier permeability plays an important role in the initiation and progression of IBD. Our results confirmed that different doses of SYD exerted significantly anti-colitis effects, repressed intestinal inflammation and restored epithelial barrier function, implicating that SYD may alleviated colitis largely by restoring epithelial barrier function.

Dysregulated epithelial cell death contributes to epithelial barrier loss in IBD. Ferroptosis is a novel programmed cell death, inhibition of ferroptosis significant alleviated chemically induced colitis [10]. Thus, we measure whether SYD restored epithelial barrier function by inhibiting ferroptosis in epithelial cells. Caco-2 cells incubated with ferroptosis inducer erastin was used to measure the effects of SYD on ferroptosis in vitro. Results showed that SYD significantly inhibited ferroptosis in Caco-2 epithelial cells, implicating the inhibition of ferroptosis contributed to SYD-induced epithelial barrier restoration and further alleviation of colitis.

Glutathione peroxidase 4 (GPX4), a selenoprotein, is the major enzyme catalysing the reduction of phospholipid hydroperoxides in mammalian cells [11]. Lipid hydroperoxides are key intermediates in the lipid peroxidation process, the reduction of lipid hydroperoxides to lipid alcohols requires the catalytic selenocysteine residue of GPX4, and this process prevents iron (Fe^2+^)-dependent formation of toxic lipid reactive oxygen species (ROS) [12]. Therefore, the reduction of GPX4 expression can lead to the accumulation of oxidized phospholipids and lipid peroxidation to promote the occurrence of ferroptosis, which has been demonstrated in animal and clinical experiments [13, 14]. Besides, GPX4 expression is decreased in chemically induced colitis and inhibition of GPX4 aggravates the pathological process of IBD [32]. In our study, colonic protein expression and mRNA level of GPX4 were all decreased after TNBS treatment, and SYD administration enhanced GPX4 expression in colitis rats. Luciferase results further confirmed that SYD may increase GPX4 expression by modulating GPX4 transcription. Knockout of GPX4 in rats further confirm that GPX4 is the target gene for SYD-induced anti-colitis effects. These suggested that SYD inhibited ferroptosis in epithelial cells and restored epithelial barrier loss in colitis in GPX4-dependent manner.

Colitis lesions are mainly limited to the mucosa, submucosa of the colon, and can diffuse to the entire colon [33]. After orally administration, drug ingredients are usually absorbed by intestinal villi and across the mucosa to the blood by passive diffusion, which then distribute to colonic tissues [34, 35]. Besides, drug ingredients that are not absorbed by intestinal villi can be absorbed by colonic epithelial mucosa, which may exert influence on colitis directly [36, 37]. To further measure the active ingredients of SYD that exerted anti-colitis effects, we measured the ingredients enriched in colonic tissues and plasma sample after SYD gastric administration. MS analysis revealed that 16 ingredients were identified in SYD, 11 ingredients and 14 ingredients were identified in plasma and colon samples from colitis rats after SYD orally administration respectively, implicating 11 ingredients may serve as the main active ingredients in SYD that exerts anti-colitis effects, although SYD is composed of hundreds of ingredients. Luciferase results showed that wogonoside, wogonin, palmatine, paeoniflorin, liquiritin exerted more significant activation of GPX4 transcription, compared with other ingredients. Macromolecular docking results proved the interaction between wogonoside, wogonin, palmatine, paeoniflorin, liquiritin and GPX4 protein. All the five ingredients in SYD exerted significant anti-colitis effects and inhibition effects of ferroptosis in epithelial cells, indicating that the five ingredients act as the key therapeutic material basis of SYD-induced anti-colitis effects based on GPX4 agonistic activity. More work plan to be done to measure more active ingredients of SYD that exerted anti-colitis effects in the near future.

## Conclusions

SYD alleviated chemically induced colitis by increasing GPX4 transcription and further inhibited ferroptosis in epithelial cells. Wogonoside, wogonin, palmatine, paeoniflorin and liquiritin served as the key therapeutic material basis of SYD-exerted anti-colitis effects. The findings provide a scientific basis for the therapeutic effect of SYD on IBD.

## Supplementary Information

**Additional file 1: Figure S1.** Identification of chemical ingredients in Shaoyao Decoction using HPLC analysis. HPLC chromatograms of Shaoyao Decoction (A) and mixture standards (B). 1: Paeoniflorin; 2: Liquiritin, 3: Ferulic acid; 4: Baicalin; 5: Berberine; 6: Emodin.

## Data Availability

The datasets used and/or analysed during the current study available from the corresponding author on reasonable request.

## Abbreviations

GPX4: Glutathione peroxidase 4
SYD: Shaoyao Decoction
TNBS: 2,4,6- Trinitrobenzene sulfonic acid
MS: Mass Spectrometry
IBD: Inflammatory bowel diseases
UC: Ulcerative colitis
CD: Crohn’s disease
DAI: Disease activity index
LPO: Lipid peroxidation
HE: Hematoxylin–eosin staining
4-HNE: 4-Hydroxynonenal
FD-4: Fluorescein isothiocyanate dextran
LPS: Lipopolysaccharide.
TEER: Transepithelial electrical resistance
TEM: Transmission electron microscopy
HPLC: High Performance Liquid Chromatography
MS: Mass Spectrometry
SASP: Sulfasalazine
