# Supplementary figures and images for: Therapeutic material basis and underling mechanisms of Shaoyao Decoction-exerted alleviation effects of colitis based on GPX4-regulated ferroptosis in epithelial cells
Source: Chin Med. 2022 Aug 16;17:96. doi: 10.1186/s13020-022-00652-1 (PMC9380349; doi:10.1186/s13020-022-00652-1)

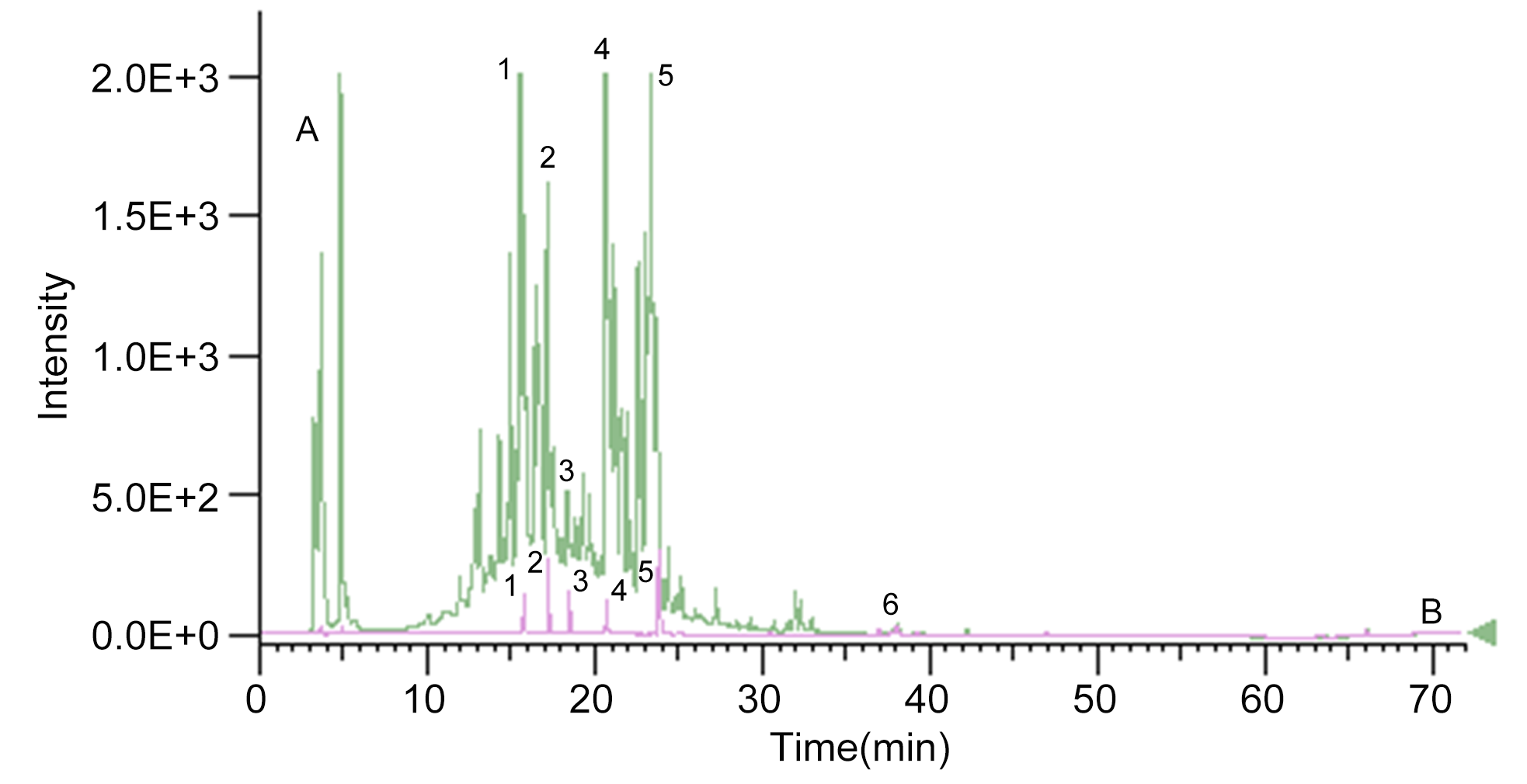

Supplement: Supplementary file 1 — Additional file 1: Figure S1. Identification of chemical ingredients in Shaoyao Decoction using HPLC analysis. HPLC chromatograms of Shaoyao Decoction (A) and mixture standards (B). 1: Paeoniflorin; 2: Liquiritin, 3: Ferulic acid; 4: Baicalin; 5: Berberine; 6: Emodin. [file 13020_2022_652_MOESM1_ESM.tif]
